# Social Media Surveillance for Outbreak Projection via Transmission Models: Longitudinal Observational Study

**DOI:** 10.2196/11615

**Published:** 2019-05-26

**Authors:** Anahita Safarishahrbijari, Nathaniel D Osgood

**Affiliations:** 1 University of Saskatchewan Saskatoon, SK Canada

**Keywords:** machine learning, infectious disease transmission, disease models, system dynamics analysis, social media, outbreaks, infodemiology, infoveillance

## Abstract

**Background:**

Although dynamic models are increasingly used by decision makers as a source of insight to guide interventions in order to control communicable disease outbreaks, such models have long suffered from a risk of rapid obsolescence due to failure to keep updated with emerging epidemiological evidence. The application of statistical filtering algorithms to high-velocity data streams has recently demonstrated effectiveness in allowing such models to be automatically regrounded by each new set of incoming observations. The attractiveness of such techniques has been enhanced by the emergence of a new generation of geospatially specific, high-velocity data sources, including daily counts of relevant searches and social media posts. The information available in such electronic data sources complements that of traditional epidemiological data sources.

**Objective:**

This study aims to evaluate the degree to which the predictive accuracy of pandemic projection models regrounded via machine learning in daily clinical data can be enhanced by extending such methods to leverage daily search counts.

**Methods:**

We combined a previously published influenza A (H1N1) pandemic projection model with the sequential Monte Carlo technique of particle filtering, to reground the model bu using confirmed incident case counts and search volumes. The effectiveness of particle filtering was evaluated using a norm discrepancy metric via predictive and dataset-specific cross-validation.

**Results:**

Our results suggested that despite the data quality limitations of daily search volume data, the predictive accuracy of dynamic models can be strongly elevated by inclusion of such data in filtering methods.

**Conclusions:**

The predictive accuracy of dynamic models can be notably enhanced by tapping a readily accessible, publicly available, high-velocity data source. This work highlights a low-cost, low-burden avenue for strengthening model-based outbreak intervention response planning using low-cost public electronic datasets.

## Introduction

The capacity to accurately project communicable disease outbreak evolution is of great value in public health planning for prevention and control strategies. Use of such information can inform resource allocation, including surge-capacity planning and planning of the timing of outbreak response immunization campaigns, and, when applied across distinct scenarios, provide a basis for evaluating tradeoffs between intervention strategies. Although dynamic models are increasingly widely used to conduct such scenario projection, the construction of such models for new and rapidly evolving pathogens commonly faces significant barriers due to uncertainties regarding important factors governing the natural history of the disease, such as duration of latent, incubation, and infectious phases; the probability of asymptomatic carriage; rates of waning immunity; contact rates; and per-discordant-contact transmission probabilities. Moreover, even the most intricate models face strict limitations in their ability to project evolution of factors treated as stochastic, such as weather-related variables and the timing of arrival of exogenous infections due to global travel. Using computational statistical estimation methods such as sequential Monte Carlo techniques, in recent years, researchers have contributed approaches to elevate the predictive accuracy of dynamic transmission models by updating their state estimates at the time of appearance of each new observation. The predictive accuracy of methods has thus far been evaluated purely in the context of models that make use of traditional surveillance data sets, such as laboratory and clinically confirmed case reports [1-6].

Although such traditional surveillance data sets offer high-quality, rich information about individuals who present for medical care, they suffer from notable shortcomings, including delayed reporting and a failure to include counts of infective individuals who choose not to present for care. In a separate stream of work from the dynamic modeling work noted above, in recent years, researchers have sought to compensate for the limitations of traditional epidemiological data sources more generally by exploiting information related to online communicational behavior, particularly, the growing tendency of many users to search, post, and tweet about their illnesses. Specifically, such researchers have assessed the health insights that can be gained from public health surveillance applications employing a variety of online sources of information.

A prominent line of this work has focused on time sequences of search query volumes, such as those previously captured in Google Flu Trends (GFT) [7] and (on a more generic and continuing basis) Google Trends [8]. Within this sphere, a wide variety of investigations have used statistical and machine learning methods to perform classification and analysis on such Google search volume data and volumes of social media postings, including those for communicable illnesses [9-12]. Many researchers have investigated biomedical and health-related knowledge obtained from the Twitter platform, suggesting opportunities and limitations associated with different machine learning classifiers and training models for tweet mining [13-15]. Other case studies have reported a significant correlation between Tweets and clinical reports and concluded that social media text mining can improve public health communication efforts by providing insight into major themes of public concerns in the health sphere [16,17].

An important subset of research in this area has leveraged data obtained from Google to develop statistical forecast models and evaluated the degree to which GFT data in combination with statistical models can support accurate predictions [18-20] and correlations with real-time empirical data [21]. Some investigators jointly used multiple data sources, including GFT and Twitter, and compared the performance of statistical prediction models using each data source and in scenarios where different data sources complement one another [22,23].

The prediction of epidemic outbreaks by dynamic models often involves significant error and generally needs to consider both underlying dynamics and noise related to both measurement and process evolution. Although older techniques based on Kalman Filtering and variants [24] have long provided a computationally frugal means of filtering stochastic dynamic models, such maximum likelihood estimation–based approaches are impaired by strong distributional assumptions concerning measurement and process noise and limited accommodation for nonlinearity in characterization of the system. This challenge in handling nonlinearity is experienced most in terms of an inability to capture the effects of probability distributions across multiple basins of attraction and a requirement for model linearization that is problematic for important modeling formalisms, such as agent-based models. For these and other reasons, recent research has increasingly turned to stronger filtering methods. Several authors have applied the sequential Monte Carlo technique of particle filtering as an effective tool in support of both model estimation and predictions from real-world data. Ong et al established a real-time surveillance system in Singapore to feed data into a stochastic model of influenza-like disease dynamics, which was refitted daily using particle filtering [1]. Osgood and Liu used a synthetic ground truth model to evaluate the effectiveness of particle filtering for an H1N1-like infection in the presence of noisy data and systematic model simplifications [2]. Safarishahrbijari et al evaluated the effectiveness of particle filtering subject to specifics of the configuration, such as frequency of data sampling and representation of behavior change in the form of an evolving contact rate for H1N1 [3,5]. Oraji et al developed a system dynamics model for studying the tuberculosis transmission and applied particle filtering to estimate the latent state of the system, including many epidemiological quantities that are not directly measured. Their results suggested an improvement in model accuracy using particle filtering and high additional value extending from consideration of additional epidemiological quantities in the probabilistic model [4]. Li et al applied particle filtering to a measles compartmental model using reported measles incidence for Saskatchewan. They also performed particle filtering on an age-structured adaptation of their model by dividing the population into age groups for children and adults. According to their results, particle filtering can offer high predictive capacity for measles outbreak dynamics in a low-vaccination context [6].

Epstein et al explored the effect of adaptive behaviors such as social distancing based on fear and contact behavior in models of epidemic dynamics. They used nonlinear dynamic systems and agent-based computation and integrated disease and fear of the disease contagion processes. Based on their models, individuals anxious (“scared”) about or infected by a pathogen can transfer fear through contact with other individuals who are not scared, and scared individuals may isolate themselves, thereby influencing the contact rate dynamic, which is a key parameter in governing outbreak evolution. The authors studied flight as a behavioral response and concluded that even small levels of fear-inspired flight can have a dramatic impact on spatiotemporal epidemic dynamics [25].

Despite the fact that both high-velocity search volume and social media data and transmission models share a temporal perspective, data drawn from such internet series has not, to our knowledge, been previously used as a source of information for filtering (via recurrent regrounding) compartmental transmission models with the arrival of new data.

In this work, we sought to address that gap by combining the transmission model from the study by Epstein et al [25] with the sequential Monte Carlo method of particle filtering, considering the interaction between disease and fear of disease contagion processes for the 2009-2010 H1N1 influenza pandemic. The particle filtered model used time series of both clinically observed data and daily Google search query volumes to automatically and recurrently reground the model as successive data points became available. Based on lessons learned from previous studies [3,5] about the importance of incorporating higher-velocity data rather than time-averaged data, we made use of daily data. In contrast to past particle filtering work on grounding transmission models, which have used empirical data purely as a comparison with model results reflecting the natural history of infection, the model presented here engaged in such comparisons for the clinical data and further compared the search query volume data with ideation-related model state (individuals with fear).

## Methods

### Particle Filtered Model

**Figure 1:**
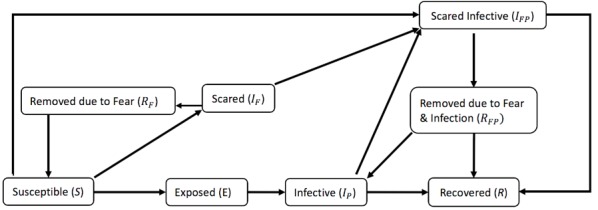
System dynamics model.

In the first stage of characterization of the particle filtered model, we present the formulation of the existing Epstein compartmental model from a previous study [25], which characterizes the population into states according to both their natural history of infection and presence of anxiety regarding influenza. The state variables of the model are as follows: Susceptible to pathogen and fear (*S*), Infected with fear (*I*_F_), Infected with pathogen (*I*_P_), Infected with pathogen and fear (*I*_FP_), Removed due to fear (*R*_F_), Removed due to fear and pathogen (*R*_FP_), and Recovered (*R*). We used an adaptation of the model that included an Exposed (*E*) state variable (Figure 1). In this model, λ_F_ is the (hazard) rate of removal due to self-isolation of those in fear only, λ_P_ refers to the rate of recovery from infection with pathogen, λ_FP_ represents the rate of removal due to self-isolation of the infected who are also afraid, and *H* is the rate of recovery from fear (alone) and return to circulation [25]. The parameters *α* and *β* denote transmissibility of fear and pathogen, respectively. Specifically, α represents the probability that a contact between an individual *A* who is currently without fear but who is susceptible or infected purely with the pathogen and an individual *B* with either fear or the pathogen will cause individual *A* to become afraid. In contrast, *β* denotes the probability that a contact between an individual *A* who has never been infected with the pathogen and an individual *B* who is specifically infected with the pathogen will infect individual *A* with the pathogen. Given that *α* and *β* are probabilities (and are thus of unit dimension), it bears emphasis that simple dimensional analysis demonstrates that the original authors assume an effective per-person-per-unit time mixing rate with a value of unity. Although not considered within the scope of the original article, this mixing rate can itself be characterized in accordance with long-time mathematical epidemiology practice as the product of a per-unit-time contact rate *c* and disease transmissibility divided by the (constant) total population *N.* Because we consider changes to the value of *c* within this work, this quantity is shown explicitly in the equations below. To explain this term, which is required for dimensional consistency, we note that each transmission term, such as: 
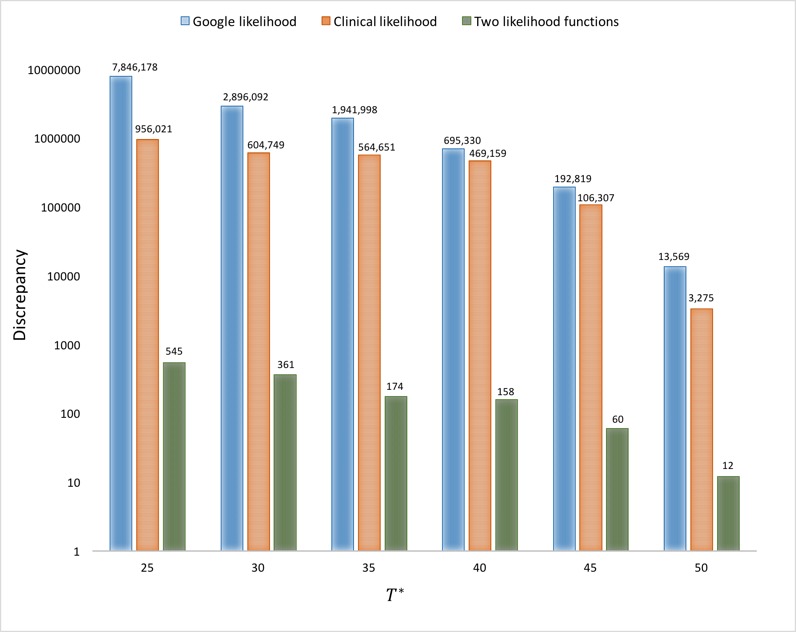
can be considered to characterize the rate of transmission (in terms of persons per unit time) from possible transmitters in category *Y* (here, *I*_FP_) to persons in at-risk category *X* (here, *S*). Each such at-risk person *X* is assumed to engage in an average of *c* contacts per unit time. Those overall contacts are then assumed to be spread proportionally among the compartments in the population, with the fraction taking place with those in a category *Y* of possible transmitters, which is the count of people in *Y* divided by the total population *N*. The probability in the prefix of the term (here, *βα*) indicates the probability that each such potentially transmitting contacts leads to the type of transmission being considered in that term (either fear, pathogen, or, as in this example, both).

When adapting the model, we took advantage of the previously demonstrated [3,5] capacity of particle filtering to support stochastic evolution of designated parameters (captured as state variables). One of the stochastic parameters included in this model represents the fraction of reported incidents (*f*_P_), which is the fraction of people who are reported to public health authorities when emerging from the latent state and is both uncertain and evolving over time. Likewise, the fraction of people becoming afraid who search Google upon infection, named the fraction of Google search incidents (*f*_F_), is further treated as a dynamic uncertain parameter.

Other parameters also treated as stochastic are the contact rate (*c*), removal rate from those with fear to self-isolation (λ_F_), and removal rate from those with fear who are also infected (λ_FP_). To support this, such dynamic parameters are associated with state variables evolving over time according to stochastic differential equations. Because variable *c* is a nonnegative quantity, we performed a log-transform on this variable according to the Brownian Motion, so that it varied over the real numbers. The stochastic differential equation of contact rate *c* is described as:


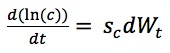
(1), where *dW*_t_ is a standard Wiener process following a normal distribution with mean of 0 and variance of 1. Thus,


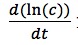


follows a normal distribution with mean of 0 and variance of *s*_c_^2^. We also performed a log-transform on λ_F_; the stochastic differential equation of λ_F_ is formulated as: 
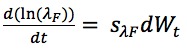


The initial values of *c* and λ_F_ are drawn uniformly from the interval between 0 and 100 per day and between 0.4 and 1 per day, respectively. The SDs of *s*_c_ and *s*_λF_ were both selected to be 1.

In contrast, reflecting the fact that *f*_P_ and *f*_F_ represent fractions, such parameters were logit-transformed, with the initial value for each varying between 0 and 0.2. We described the stochastic differential equations of fractions *f*_P_ and *f*_F_ according to Brownian Motion as: 
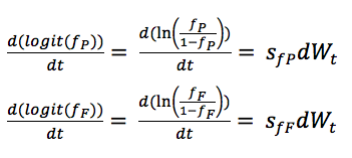


Within the model, the parameter *f*_P_ is multiplied by inflows to state variables Infective (*I*) and Scared Infective (*I*_FP_) to account for fractional actual reporting. Similarly, the parameter *f*_F_ is multiplied by inflows to state variables Scared (*I*_F_), Scared Infective (*I*_FP_), Removed due to Fear and Infection (*R*_FP_), and Removed due to Fear (*R*_F_) and accounts for the fractional of the actual scared population.

We treated λ_F__P_ as: 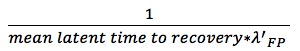
and then considered λ'_FP_ as a fraction and performed a logit-transform on it. This parameter varies over the range from 0 to 1 and the dynamic process for λ'_FP_ is similar to *f*_P_ and *f*_F_, specifically,


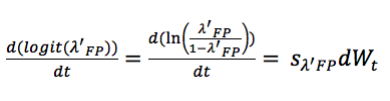


The SDs *s*_fP_, *s*_fF_, and s_λ'FP_ are selected to be 5, 5 and 1, respectively. The initial values of *f*_P_, *f*_F_, and λ'_FP_ are set on the intervals [0, 0.2), [0, 0.2) and [0, 0.5), respectively.

By applying random walks to these parameters, a more accurate estimate was achieved during model simulation. As such, in our model, each particle at each point in time is associated with all state variables and state variables associated with stochastic parameters (*S*, *E*, *I*_F_, *I*_P_*, I*_FP_, *R*_F_, *R*_FP_, *R*, *c*, *f*_P_, *f*_F_, λ_F_, and λ'_FP_) (Table 1).


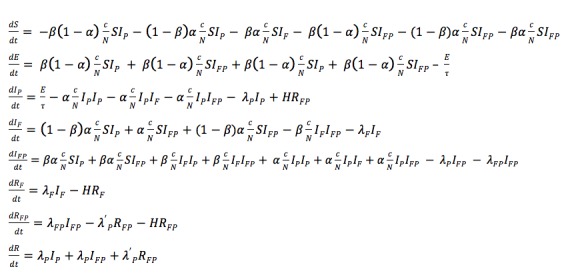


**Table 1:** Parameters used in the model.

Parameter name	Notation	Value for Quebec	Value for Manitoba	Unit
Probability of infection transmission given exposure	β	0.04	0.04	Unit
Probability of fear transmission given exposure	α	0.02	0.02	Unit
Mean latent time	τ	3	3	Day
Mean time to recovery	μ	7	7	Day
Total population of province	*N*	7843475	1214403	Person
Rate of recovery from fear	*H*	0.2	0.2	One per day
Rate of removal to self-isolation from fear	λ_F_	Dynamic	Dynamic	One per day
Fraction of mean time to recovery of going from “Scared Infected” to “Recovered” via “Removed Due to Fear & Infection”	λ'_FP_	Dynamic	Dynamic	Unit
Rate of removal to self-isolation from fear and pathogen	λ_FP_	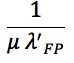	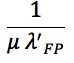	One per day
Rate of recovery from infection with pathogen	λ_P_	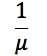	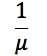	One per day
Rate of recovery from removal due to fear and infection	λ'_P_	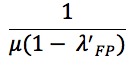	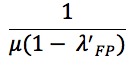	One per day

### Description of Data Sources

We evaluated the prediction of the above-described dynamic model assisted by particle filtering against two publicly available empirical datasets. The first was from Manitoba Health - Healthy Living and Seniors and included daily laboratory-confirmed case counts of pandemic H1N1 influenza for the period of October 6, 2009, through January 18, 2010, for the province of Manitoba [26]. The second dataset was from the Institut National de Santé Publique du Québec, a public health expertise and reference center in Quebec, and included daily confirmed case counts of pandemic H1N1 influenza between October 6, 2009, and December 19, 2010 [27].

In addition to the daily clinical case count data noted above, we obtained normalized daily Google search counts from Google trends and weekly normalized data from GFT for Manitoba and Quebec during the second pandemic wave. Reflecting the linguistic differences between the two provinces, the search terms used for each were distinct. In Manitoba, we used search terms “flu” and “H1N1,” while for Quebec, we used “flu,” “Influenza A virus sub-type H1N1,” “h1n1 vaccination,” “ah1n1,” “ah1n1 vaccin,” “grippe,” and “grippe ah1n1,” which are the most frequent search queries related to this topic suggested by Google during that period.

### Particle Values and Parameter Values

When defining the likelihood function for observing empirical data, given the state of a given particle, the exact variant of the likelihood used varied across three different scenarios examined. The first scenario evaluated the impact of assuming a likelihood formulation that considered purely clinical data, termed *L*_infection with pathogen_. The likelihood being used in the second scenario considered only the likelihood of observing the empirical data regarding Google search counts for the appropriate province in light of the count of individuals posited to be currently in fear within the model, a likelihood denoted as *L*_infection with fear_.

Following several past contributions [2-4,28], we assume that each epidemiological quantity follows a Pascal distribution function. Thus, *y*_t_ and *i*_t_ represent observed individuals per day and particle-posited daily rate (count per day) of new cases, respectively: 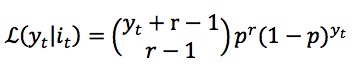
In the formulation for the likelihood function, *r* is a dispersion parameter


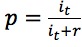
and 
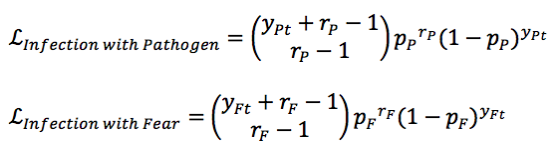
where *y*_Pt_ and *y*_Ft_ represent number of laboratory-confirmed incident cases reported for day *t* and number of Google search incidents for that day, respectively. The probabilities *p*_P_ and *p*_F_ follow 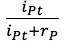
and 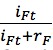
respectively, where *i*_Pt_ is a fraction of the flow of new cases of infection and *i*_Ft_ is a fraction of the flow of new cases of scared. The dispersion parameter *L*_infection with pathogen_ (*r*_P_) was considered as 40, while *L*_infection with fear_ (*r*_F_) was considered as 25. This reflects the larger noise that we believed to be associated with Google search data, in light of the fact that a larger dispersion parameter leads to a more narrowly dispersed distribution.

The third scenario considered a total likelihood function *L*_T_ consisting of a combination of *L*_infection with pathogen_ and *L*_infection with fear_. For defining the total likelihood function, the simplifying assumption was made that deviations with respect to one measure were independent of the other, and thus, the total multivariate likelihood function could be treated as a multiplication of two univariate likelihood functions, given as *L*_T_=*L*_infection with pathogen__×_*L*_infection with fear_>

The purpose of running this third scenario was to compare the effectiveness of a univariate likelihood function with that of the multivariate likelihood function, when evaluated in terms of a calculated discrepancy of model predictions against the epidemiologically confirmed case count.

The three scenarios noted above were conducted using particle filtering, employing 1000 particles. For each such scenario, reflective of the need to make decisions in light of uncertainty about the evolution of an unfolding outbreak, in which only information about time points up to the present is available, we sought to examine the impact of right censoring the empirical data at certain time point T*, representing the current time (ie, the time from which the model is forecasting outbreak evolution). Thus, as the model ran, particle weights were updated based on observations from day one until and including day T*; after day T*, particle filtering ceased, particle weights were no longer updated using historic data, and no further particles were resampled. Each scenario included a sequence of subscenarios that employed the following distinct values of T*: {25, 30, 35, 40, 45, 50}.

To judge the accuracy of particle filter *–* informed projections for future times against the standard of the reported case counts for those times, we defined a discrepancy metric as the expected value of the *L*^2^ norm of the difference between sampled particles (reporting rate coefficient × [infected state+scared infected state]) and reported case count observations calculated after time T*. We sampled n particles (n=700) according to their weights and obtained the discrepancy value using the following equation: 
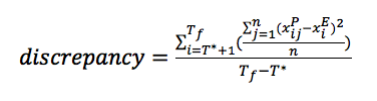
where 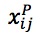
is the value associated with sampled particle *j* at observation *i* and 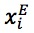
is the respective reported clinical cases at observation *i*. *T*_f_ is the final observation time, and T* indicates the time from which the projection is being made (ie, the time up to which the particles’ weights were updated based on observation, where 0 ≤ t ≤ T*). Using this formulation, we evaluated how well projections forward predicted the empirical data after T*, the time at which particle filtering was completed.

## Results

In this work, for each scenario (each associated with a particular likelihood function), we plotted the graphs associated with T*=30 for Manitoba and Quebec. We characterize the results below according to the scenario.

### Particle Filtering Using Two Likelihood Functions

**Figure 2:**
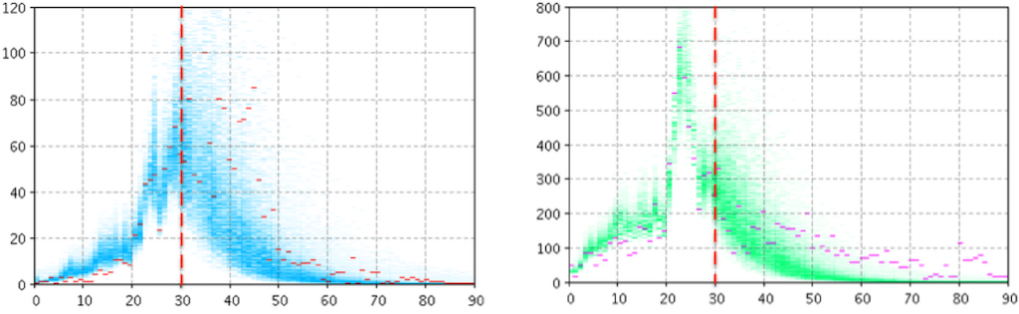
Empirical data (red and magenta points) superimposed on samples (blue and green) from the model-generated distribution of particles for the model output of the count of reported cases (left panel) and number of searches (right panel) using two likelihood functions, T*=30 for Manitoba.

**Figure 3:**
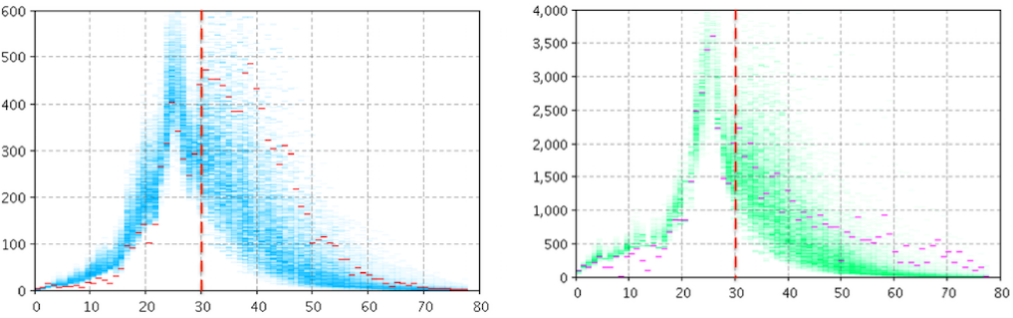
Empirical data (red and magenta points) superimposed on samples (blue and green) from the model-generated distribution of particles for the model output of the count of reported cases (left panel) and number of searches (right panel) using two likelihood functions, T*=30 for Quebec.

Figures 2 and 3 depict the empirical data (red and magenta points) superimposed on samples (blue and green) from the model-generated distribution of particles for the model output of the number of reported cases (left panel) and number of searches (right panel) for Manitoba (Figure 2) and Quebec (Figure 3). For T*=30, the high posterior density for the projection period is quite localized for the cases of pathogen and the number of searches.

### Particle Filtering Using the Likelihood Function Associated With Clinical Data Alone

**Figure 4:**
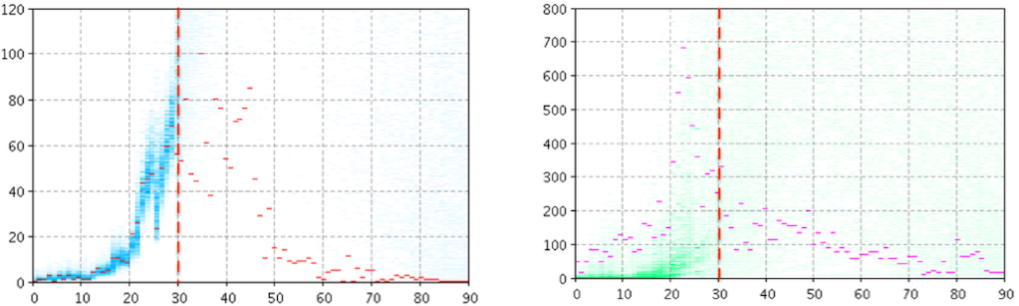
Empirical data (red and magenta points) superimposed on samples (blue and green) from the model-generated distribution of particles for the model output of the count of reported cases (left panel) and count of searches (right panel) using the likelihood function associated with clinical data alone, T*=30 for Manitoba.

**Figure 5:**
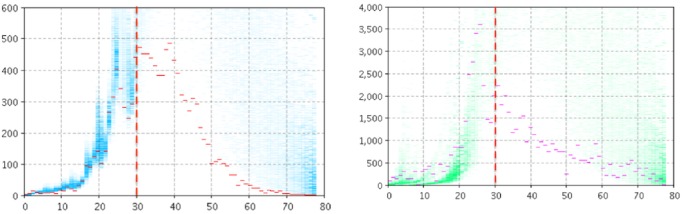
Empirical data (red and magenta points) superimposed on samples (blue and green) from the model-generated distribution of particles for the model output of the count of reported cases (left panel) and count of searches (right panel) using the likelihood function associated with clinical data alone, T*=30 for Quebec.

In this configuration, particle filtering was performed using *L*_infection with pathogen_ as the sole likelihood function. Figures 4 and 5 depict empirical data (red and magenta points) superimposed on samples (blue and green) from the model-generated distribution of particles for the model output of the number of reported cases (left panel) and number of searches (right panel) for Manitoba (Figure 4) and Quebec (Figure 5). Despite the fact that the particle filtering employs reasonably high-resolution clinical data, the system exhibits great difficulty both in accurately projecting the number of clinical case reports forward from the point where particle filtering ceases (T*) and in doing so in a fashion where the high posterior density region is localized. Unsurprisingly, the model informed by the reported clinical case counts alone is unable to accurately characterize the search volume within the population.

### Particle Filtering Using the Likelihood Function Associated With Search Volume Data Alone

**Figure 6:**
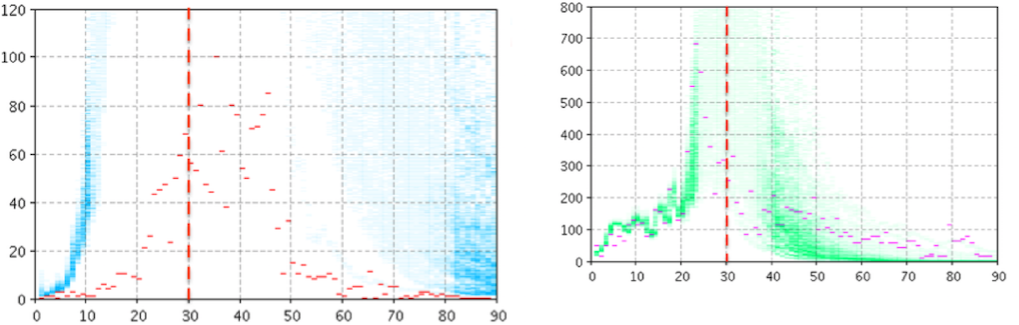
Empirical data (red and magenta points) superimposed on samples (blue and green) from the model-generated distribution of particles for the model output of the count of reported cases (left panel) and count of searches (right panel) when using the likelihood function associated with search volume data alone, T*=30 for Manitoba.

**Figure 7:**
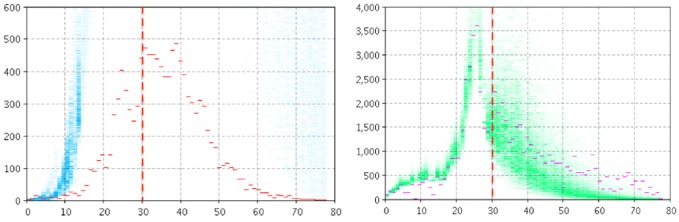
Empirical data (red and magenta points) superimposed on samples (blue and green) from the model-generated distribution of particles for the model output of the count of reported cases (left panel) and count of searches (right panel) when using the likelihood function associated with search volume data alone, T*=30 for Quebec.

In this configuration, particle filtering was performed using *L*_infection with fear_ as the sole likelihood function. Figures 6 and 7 depict empirical data (red and magenta points) superimposed on samples (blue and green) from the model-generated distribution of particles for the model output of the number of reported cases (left panel) and number of searches (right panel) for Manitoba (Figure 6) and Quebec (Figure 7). Although the results for both jurisdictions show some localization in the projections of the prevalent case count of those living in fear, the failure to consider the clinical case count in particle filtering (and to accordingly update the model estimates for the current number of infectives, susceptibles, and the contact rate) leads to poor projection accuracy for the reported clinical case count.

**Table 2:** Discrepancies associated with different scenarios and T* values for Manitoba and Quebec.

Scenario		T*									
		25	30	35	40	45	50
**Manitoba**											
	Google Likelihood	7,846,178	2,896,092	1,941,998	695,330	192,819	13,569
	Clinical Likelihood	956,021	604,749	564,651	469,159	106,307	3275
	Two Likelihoods	545	361	174	158	60	12
**Quebec**											
	Google Likelihood	577,919,468	437,577,329	290,486,216	108,993,972	29,645,905	9,179,791
	Clinical Likelihood	31,571,941	3,544,611	461,804	55,938	4862	751
	Two Likelihoods	535,927	17,386	8338	3322	1071	336

### Comparison of Results Associated With Different Scenarios

Table 2 depicts the discrepancies between model clinical case predictions and empirical data for different check times (T*) for Manitoba and Quebec. Unsurprisingly, given the results above, the discrepancy associated with particle filtering informed by both clinical and search volume data sets (in Particle Filtering Using Two Likelihood Functions) is smaller than the discrepancy associated with either data set in isolation. In addition, the discrepancy when using particle filtering informed by the (higher-quality) clinical case count data alone is lower than that informed purely by search volume. However, there is a marked difference between Manitoba and Quebec in the levels of discrepancy seen when using clinical case data alone as compared to using search volume data. For Manitoba, there is consistently less than an order of magnitude of difference in discrepancies between these two results. In contrast, for Quebec, using the clinical data alone within particle filtering yields a level of discrepancy several orders of magnitude below that resulting from search volume data. Intriguingly, for Manitoba, combining both data yields a reduction of discrepancy many orders of magnitude below either, despite the fact that discrepancy is calculated with respect to clinical case reports. This advantage of adding information from the search volume data to that from clinical case counts presumably reflects the fact that the added search volume information supports particle filtering in more accurately localizing the model state estimates than was the case using purely the reported clinical case counts—a factor manifested in the projections for both clinical case counts. In contrast, for Quebec, using both sources of information reduces the discrepancy significantly, typically by at least one order of magnitude, with the exception of time points T*=45 and T*=50.

## Discussion

### Principal Findings

In this contribution, we investigated the predictive accuracy gains from applying particle filtering using both traditional and search volume data to estimate latent states of a compartmental transmission model (including time evolution of stochastic parameters involved in that model). The capacity to perform this estimation then provides support for projection and scenario evolution using the model.

To be able to use search data effectively when particle filtering a transmission model, we found it helpful to move beyond the traditional scope of compartmental transmission models and to adopt a more articulated model of the outbreak, reflecting the fact that causal drivers promoting Web searches are not restricted to stages in the natural history of infection, but are additionally driven by factors with distinct but coupled dynamics, such as fluctuations in perceived risk on the part of the population. Responsive to this consideration, we have adapted a previously published model with an explicit consideration of the coupled dynamics of fear and pathogen. Although there are challenges associated with assessing perceived risk and anxiety on the part of the population during an outbreak, we found here that projection of outbreak dynamics can be materially enhanced through inclusion of a surprisingly accessible source of data: Daily relative search query volumes for defined geographic regions on the widely used Google search engine. The reliable and timely public availability of such data across many areas of the world raises the prospects for significantly enhancing effective outbreak projection using combinations of dynamic modeling and machine learning techniques such as the particle filter.

### Limitations

The work presented here has significant limitations. Although search trend data provide some indication of topic-specific interest over time in a defined spatial region, from the standpoint of “big data,” it is often available only with modest (daily) temporal resolution and frequently coarse geographic resolution. It is also affected by many unobserved confounders. Such search trend data are further limited by providing little sense of count of distinct users and no sense of longitudinal progression of a single user. In this regard, the Google search query volume time series compare unfavorably to the richness of information present in other publicly available types of online data, such as region-specific Twitter feeds.

In addition to the shortcomings in the data sources employed, there are notable methodological limitations of our study. The likelihood function employing two distinct data sources was simplistic in its design, merely serving to multiply each of the dataset-specific likelihood functions. The use of a random walk during particle filtering for no fewer than five distinct parameters likely contributes to a rapid divergence in the model’s estimates, compared to the behavior observed in previous particle filtered models of influenza [1,3]. Further experimentation is required with the parameters governing such random walks. A more significant yet large gain in accuracy, given the limited volatility likely for some of such parameters, may result from treating such parameters as unknown constants to be sampled for a given simulation from a posterior distribution within Particle Markov Chain Monte Carlo (PMCMC) techniques [29].

Such limitations point to natural avenues for future work. We expect that the prospects for the sorts of projections explored here will be significantly elevated by combining such data with other public data sources containing distinct sources of information, such as daily or finer resolution time series from Twitter and Tumblr. We further expect the accuracy of the projections to be improved by more powerful machine learning techniques, such as through the use of PMCMC techniques, ensemble techniques supporting inclusion of multiple models, and potential PMCMC techniques employing multiple models using reverse-jump MCMC strategies.

### Conclusions

Pandemic forecasting is important for public health policy making due to its support for judicious planning involving resource allocation. Official statistics typically capture only subsets of the epidemiological burden (eg, the subset of individuals who engage in care seeking). Prospects for rapid use of such data to understand outbreak evolution are often further handicapped by reporting delays and a lack of capacity to project epidemiological case count time series forward. Traditional outbreak data have been complemented in recent years by high-resolution data sets from public social media such as Twitter, Tumblr, and time series provided by the Google search application programming interface via Google trends and Google flu, which can be retrieved programmatically and analyzed over time. The results presented in this work suggest that, when combined with traditional epidemiological data sources, social media–driven data sets, machine learning, and dynamic modeling can offer powerful tools for anticipating future evolution of and assessing intervention tradeoffs with respect to infectious disease outbreaks, particularly for emerging pathogens.

## Abbreviations

GFT: Google Flu Trends
PMCMC: Particle Markov Chain Monte Carlo
